# Acute physiological, biomechanical, and perceptual responses of runners wearing downward-curved carbon fiber insoles

**DOI:** 10.3389/fspor.2024.1340154

**Published:** 2024-04-05

**Authors:** Florian A. Engel, Frank Zehnter, Tomer Yona, Patrick Mai, Steffen Willwacher, Peter Düking, Billy Sperlich

**Affiliations:** ^1^ Integrative and Experimental Exercise Science and Training, University of Würzburg, Würzburg, Germany; ^2^ Department of Biomedical Engineering, Technion Israel Institute of Technology, Haifa, Israel; ^3^ Department of Mechanical and Process Engineering, Offenburg University of Applied Sciences, Offenburg, Germany; ^4^ Department of Sports Science and Movement Pedagogy, Technische Universität Braunschweig, Braunschweig, Germany

**Keywords:** footwear, running shoes, shoe technology, running economy, running performance

## Abstract

In a randomized controlled cross-over study ten male runners (26.7 ± 4.9 years; recent 5-km time: 18:37 ± 1:07 min:s) performed an incremental treadmill test (ITT) and a 3-km time trial (3-km TT) on a treadmill while wearing either carbon fiber insoles with downwards curvature or insoles made of butyl rubber (control condition) in light road racing shoes (Saucony Fastwitch 9). Oxygen uptake, respiratory exchange ratio, heart rate, blood lactate concentration, stride frequency, stride length and time to exhaustion were assessed during ITT. After ITT, all runners rated their perceived exertion, perceived shoe comfort and perceived shoe performance. Running time, heart rate, blood lactate levels, stride frequency and stride length were recorded during, and shoe comfort and shoe performance after, the 3-km TT. All parameters obtained during or after the ITT did not differ between the two conditions [range: *p *= 0.188 to 0.948 (alpha value: 0.05); Cohen's d = 0.021 to 0.479] despite the rating of shoe comfort showing better scores for the control insoles (*p *= 0.001; d = −1.646). All parameters during and after the 3-km TT showed no differences (*p *= 0.200 to 1.000; d = 0.000 to 0.501) between both conditions except for shoe comfort showing better scores for control insoles (*p *= 0.017; d = −0.919). Running with carbon fiber insoles with downwards curvature did not change running performance or any submaximal or maximal physiological or biomechanical parameter and perceived exertion compared to control condition. Shoe comfort is impaired while running with carbon fiber insoles. Wearing carbon fiber insoles with downwards curvature during treadmill running is not beneficial when compared to running with control insoles.

## 1 Introduction

Between 2014 and 2024 long distance running performance in male and female runners has improved (1, 2). In recent years, both male and female marathon world records have witnessed significant advancements, marked by improvements of several minutes (1, 2). The marathon world record for male runners between 2007 and 2014 was improved four times by an overall of 89 s, each new marathon world record showed an improved running time of 15–27 s. Whereas the progression from one world record to the next from 2014 (2:02:57, h:min:s, Dennis Kimetto, Berlin 2014) to 2018 (2:01:39 h:min:s, Eliud Kipchoge, Berlin 2018) saw an improvement of 78 s. Numerous investigations offer a wide range of explanations for the improvement in distance running including (but not exclusively) increased training volume and intensity (2), improved pacing and drafting tactics, as well as optimized carbohydrate feeding (2).

Advancements in running shoe technology might also be a component for the ergogenic developments in running performance since the decline in running times coincidences with the appearance of road running shoes consisting of a resilient, high-energy returning midsole foam and an integrated carbon fiber plate (3, 4) in 2017, the Nike Vaporfly Elite (Nike Inc., Beaverton, United States). The new generation of road running shoes are lighter than other road racing shoes and the integrated carbon fiber plate increases the longitudinal bending stiffness of the shoe (3, 4).

Submaximal running with the novel running shoe (5–8) required 3%–4% less oxygen consumption (i.e., a surrogate for improved running economy) in highly trained (5–7) and trained runners (8) translating to approximately 1% improvement in running performance (2, 9). Together with other important variables such as peak oxygen uptake and running speed at blood lactate thresholds (10–12) running economy represents an important physiological component of long-distance running performance.

Improvement in long distances performance with such new running shoe technology are not exclusively limited to elite runners. Recreational long-distance runners, when wearing the Nike Vaporfly or Nike Next%, revealed a ∼73% to 75% chance of running the (half-) marathon 4%–5% faster and achieving a new personal best when compared to running with conventional road running shoes (13). The latter analysis is based on an extensive data analysis of the social network Strava (Strava, San Francisco, USA) suggesting the improvement in running times is linked to the development of shoe technology. The latter analysis did not undergo a rigorous peer-review process, making it challenging to ascertain whether scientific standards were adhered to.

A recent systematic review with meta-analysis, including twelve studies with 188 runners revealed improved running economy while running with shoes with permanently integrated carbon plates (causing an increased longitudinal bending stiffness) when compared to conventional running shoes (14). The average improvement in running economy was 2.2% for all studies, while upwards curved carbon plates exhibit slightly greater improvements of 3.5% (14). The improved running economy was accompanied by improved step length and ground contact time (14). All of the published studies addressed upwards (facilitating toe dorsiflexion) curved carbon elements, which were integrated permanently within the shoe, however a downward curved stiffening element might induce an earlier bending of the carbon element and thus change the amount and timing of energy storage and return at the metatarsophalangeal joints (15). Furthermore, because of the downwards curved geometry, higher stiffness might be achieved with less carbon fiber material, allowing overall less shoe mass. However, so far, no study has addressed the effects of a carbon fiber element with a special downward curved design on selected variables of running physiology and biomechanics.

In many studies, the increased longitudinal bending stiffness of running shoes was obtained by upwards curved carbon plates permanently fabricated into the sole of the running shoe. The concept of removable carbon fiber plates, used as a shoe insole, aiming to increase the longitudinal bending stiffness of the running shoe and with that the running economy and ideally running performance, is relatively new. Only a few studies have assessed this concept to analyze the effects of altered bending stiffness on running biomechanics (16, 15, 17). Compared to permanently integrated carbon fiber plates exchangeable carbon plates allow runners to use different shoe brands. For recreational runners the exchangeable insoles in different shoes seems beneficial, especially to identify shoes with elevated running comfort.

The present study aimed to compare running economy and maximal running performance as well as selected physiological, biomechanical, and perceptual variables during incremental treadmill running and a 3-km time-trial (TT) in male trained runners wearing either a pair of carbon fiber insoles with a special downwards curved design or control insoles in road racing shoes. Based on the results of recent studies, we hypothesized that wearing downwards curved carbon fiber insoles would result in improved running economy and 3-km time-trial performance.

## 2 Materials and methods

### 2.1 Participants

Ten male trained runners volunteered for the present study. Detailed information about the runners' anthropometric and training data are summarized in Table 1.

**Table 1:** Means ± SD of anthropometric parameters, training volume and training history of the recreational runners (n = 10).

Parameter	Mean	Range
Age [years]	26.7 ± 4.9	21–34
Body height [m]	1.83 ± 6.2	1.69–192
Body mass [kg]	76.3 ± 5.7	64.2–83.3
Body Mass Index [kg/m^2^]	22.7 ± 1.3	20.7–24.3
Shoe size [EU shoe size standard; a.u.]	45.4 ± 1.3	43–46.5
Inseam length [cm]	86.0 ± 3.5	78–91
Calf width [cm]	37.5 ± 1.6	33.5–39.1
Foot strike pattern	Heelstriker: 50% (*n* = 5) Midfoodstriker: 50% (*n* = 5) Forefoot runner: 0% (*n* = 0)	n.a.
Training sessions per week [*n*]	3.5 ± 0.8	2–5
Hours running training per week [h]	3.5 ± 0.9	1.5–5
Volume of running per week [km]	35.3 ± 14.3	12.5–60
Accumulated years of running training [years]	9.4 ± 5.0	1.5–18
Recent 5-km time [min:s]	18:37 ± 1:07	16:32–20:00

Runners were recruited through social media and local running clubs. The inclusion criteria were: male non-smokers, present 5-km run time of approximately 16–20 min within the past 3–4 months. Exclusion criteria were: present or recent (<3 months) musculoskeletal injuries or acute illness prohibiting performance testing.

### 2.2 Experimental overview

In this randomized-controlled cross-over study, we analyzed the effects of wearing a downwards curved carbon fiber insole vs. a control insole during incremental treadmill running and a 3-km TT on selected physiological, biomechanical, and perceptual parameters and maximal running performance. All runners reported to the laboratory five times. During the initial laboratory visit, baseline measures (e.g., anthropometric data, training volume and history, inclusion and exclusion criteria) were obtained, proper shoe fit was ensured, and each runner was familiarized with the treadmill by running 10 min on the treadmill at a self-chosen pace.

During the initial laboratory session, all runners received a pair of light (195 ± 4.5 g per shoe) road racing shoes, the Saucony Fastwitch 9 (Saucony, Waltham, Massachusetts, U.S.), a pair of downwards curved carbon fiber insoles (31.4 ± 1.8 g per insole), own design and not available for consumer purchase, and a pair of control insoles made of butyl rubber, matched for the weight (31 ± 3 g per insole) of the carbon fiber insole.

After a habituation period of 14 days including 3 training sessions, runners performed four laboratory sessions while either wearing the carbon fiber insole or the butyl rubber insole (in a randomized order), involving two incremental treadmill tests until voluntary exhaustion and two 3-km time trials (3-km TT) on the treadmill. Before each of the four laboratory sessions, the downwards curved carbon fiber insole and the control insole were randomly assigned to the participants. The four laboratory sessions were performed with the same running shoe, the Saucony Fastwitch 9. The laboratory running sessions were performed within 18 days, at least 48 h apart, to guarantee adequate recovery between each session. All procedures were performed without any external encouragement and the researcher was alone with the respective runner. All runners performed each laboratory sessions at the same time of day and were asked to replicate their nutritional and sleep behavior, and training patterns before each session. All runners were instructed to wear the same clothing for the five laboratory sessions. The laboratory ambient conditions were preset and controlled (temperature: 18°C–21°C, humidity: 38%–50%).

### 2.3 Footwear and insole characteristics

**Figure 1:**
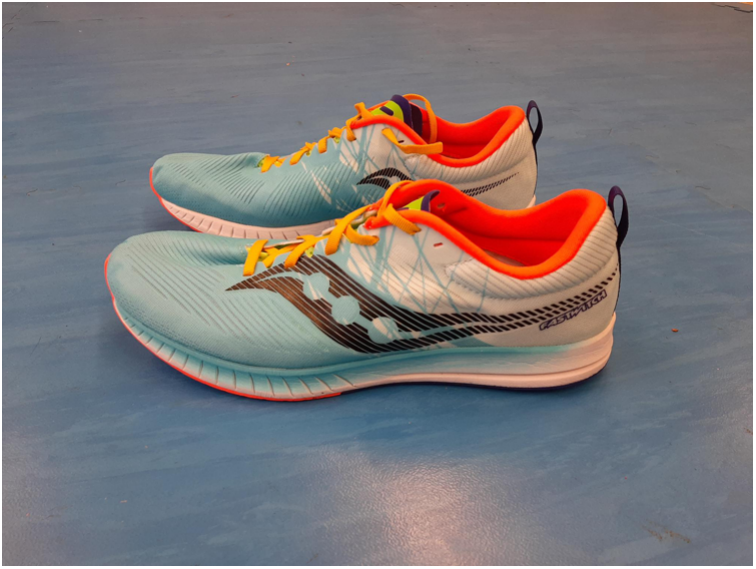
Saucony fastwitch 9 used in the study during all incremental treadmill tests and all 3-km time trials.

During the initial laboratory visit, each runner's shoe size was measured, and all runners received a new pair of the Saucony Fastwitch 9 (Figure 1). The Saucony Fastwitch has no embedded carbon fiber plate, and the midsole is made from ethylene-vinyl acetate (EVA) foam. Each runner received a written protocol about how to familiarize with the Saucony Fastwitch 9 (90 min of running, split into three sessions). After the habituation period, each runner performed all laboratory sessions with the Saucony Fastwitch 9. The basic characteristics of the running shoe are provided in Table 2. Additionally, a more comprehensive analysis of the Saucony Fastwitch 9 characteristics was performed with the minimalist index (18) (Table 3) and completed by the same rater (FAE). The minimalist index is a valid and reliable tool to determine the amount of minimalism of shoes which is explained in detail elsewhere (18).

**Table 2:** Means ± SD of the basic shoe characteristics of the participants’ (*n* = 10) running shoes (saucony fastwitch 9).

Characteristics	Saucony fastwitch 9
Mass per shoe [g]	195 ± 4.5
Stack height [mm]	18.0 ± 0.0
Heel-to-toe drop [mm]	4.0 ± 0.0

Data from the right shoe only (size: EU shoe size 43–46.5).

**Table 3:** The minimalist index with all items, subscores and the total minimalist index of the saucony fastwitch 9, size 46.5.

Characteristics	Saucony fastwitch 9	Score in the minimalist index
Mass per shoe [g]	195	3
Stack height [mm]	18.0	3
Heel-to-toe drop [mm]	4.0	4
Motion control and stability technologies [a.u.]	–	3
Longitudinal flexibility [a.u.]	–	2.0
Torsional flexibility [a.u.]	–	2.0
Minimalist index [%]	–	64

Items of the minimalist index were measured and completed by the investigator FAE. Data from the right shoe only (size: EU shoe size 46.5). Minimalist index range: 0% (lowest) to 100% (highest) degree of minimalism.

**Figure 2:**
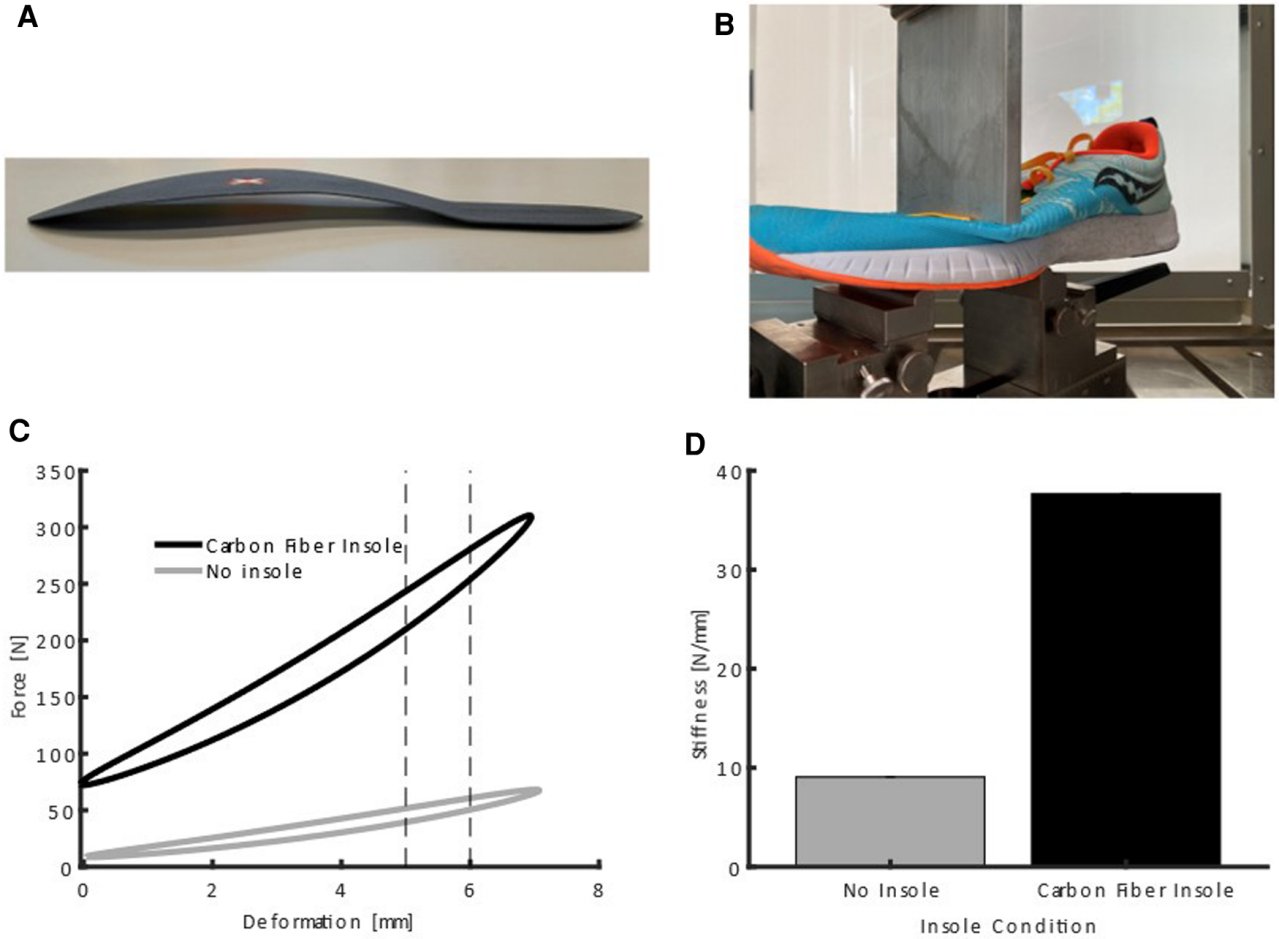
Downwards curved carbon insole (**A**) footwear used in this study in the three-point bending test apparatus (**B**) force deformation curves of the no insole and carbon insole condition (**C**) note the offset in force values resulting from the downwards curved shape of the insole. Average bending stiffness values between 5 mm and 6 mm deformation (**D**).

Additionally, all runners received a pair of carbon fiber insoles, designed with a downwards curvature, which are unavailable for purchase. The downward curved carbon fiber insoles weighed 31.4 ± 1.8 g. Each carbon fiber insole covered the entire running shoe length (Figure 2) and was manufactured according to the shoe size of each runner. A pair of butyl rubber insoles served as the control condition and were matched for mass (mean weight: 31.5 ± 0.7 g) and look of the carbon fiber insoles. To habituate to both insoles, the runners completed 45 min of running with a pair of the insoles each. Bending properties were characterized with an adapted version of a three-point bending test, as previously used (16, 15, 19). We tested the entire shoe, including the insoles, by placing them on two supporting edges 0.08 m apart. We employed a material testing machine (LTM10, Zwick GmbH & Co. KG, Ulm, Germany) to displace the combined shoe and insole at the position of the first metatarsal-phalangeal joint over a range of 7.5 mm vertically at a speed of 15 mm/s. Stiffness was calculated as the mean force required to displace the stamp from 5 to 6 mm (see Figure 2). Since the downwards curved insole already bends when the foot is positioned flat on the ground, we adapted the standard three-point bending test. We first tested the shoe with the control insoles. To determine the zero-displacement position, the material testing stamp was slowly moved until 20 N force was applied to the shoe, including the insoles and upper. The same zero position was then used to test the shoe with the downwards curved insole, resulting in a higher force at the zero position (see Figure 2).

The combined stiffness of the shoe and control insole was 9.07 ± 0.01 N/mm and 37.65 ± 0.05 N/mm for the shoe and downwards curved carbon fiber insole.

### 2.4 Incremental treadmill step test

All runners performed an incremental step test on a motorized treadmill (Pulsar®3p, h/p/ cosmos sports & medical GmbH, Nussdorf-Traunstein, Germany). The initial running speed was 10 km/h (with 1% incline) and increased by 2 km/h every 5 min until volitional exhaustion. Volitional exhaustion of each runner was verified when two of the four criteria were met (20): (i) plateau in oxygen uptake (i.e., an elevation of ≤1.0 ml min^−1^ kg^−1^ despite increasing running speed); (ii) a respiratory exchange ratio >1.1; (iii) heart rate within 5% of the age-predicted peak heart rate; (iv) self-reported rating of perceived exertion >18 on the 6–20 Borg scale (21).

Before the start, runners were instructed to run the incremental treadmill test until volitional exhaustion, respectively, to provide a maximal effort. Meta-analytical data suggests high reliability of incremental treadmill tests for trained and well-trained athletes (22).

Oxygen uptake (VO_2_), respiratory exchange ratio (RER), and ventilation (*V_E_*) (Cortex Metamax® 3B, Cortex Biophysik, Leipzig, Germany) were continuously measured throughout the treadmill testing with a metabolic cart and averaged for each 5-min increment. Before each incremental treadmill test, the metabolic cart was calibrated according to the manufacturer's guidelines. Heart rate (HR) was continuously measured with a heart rate monitor and a chest strap (Polar Vantage M2, Polar Electro© Oy, Kempele, Finland). Capillary blood samples were taken from the right earlobe at rest and immediately after each 5-min increment as well as 1 and 3 min after test termination and directly analyzed (Lactate Pro 2, Arkray KDK, Japan) to measure blood lactate. At the same time points of blood sampling all runners rated their perceived exertion (RPE) employing the Borg 6–20 scale (21). All runners were instructed how to use Borg's 6–20 scale beforehand. Stride frequency and stride length were continuously measured during the incremental treadmill test with the Polar Stride Sensor (Polar Stride, Polar Electro© Oy, Kempele, Finland) attached to the left running shoe. The Polar Stride sensor measurements are based on inertial sensors. All data of the Polar Stride Sensor were averaged for each 5-min increment. Stride length (SL) was defined as the distance between successive ground contacts of the same foot, and stride frequency (SF) as the number of ground contact events of the same foot per minute.

As described elsewhere (23), runners' foot strike pattern was analyzed by two researchers (FE and PD) from video recordings (50 Hz) while running at 70% of the speed of the last fully completed step during the incremental treadmill step test performed with the control insole.

All runners rated the shoe comfort (shoe with the respective insole) after termination of the treadmill step test (24, 8). Additionally, all runners rated the perceived shoe performance, i.e., the perceived ergogenic assistance by shoe and the insole (8) and rated their comfort and performance perception with a visual analog scale (VAS). Concerning shoe comfort, the corresponding anchor points were 0 = “Not comfortable at all” to 10 = “Maximal comfort” and for shoe performance: 0 = “no perceived support of performance during running” to 10 = “Support of performance while running” (8, 24).

### 2.5 3-km time-trial on the treadmill

All runners performed a 3-km time trial on a motorized treadmill (3-km TT) (Pulsar®3p, h/p/ cosmos sports & medical GmbH, Nussdorf-Traunstein, Germany) to assess maximal running performance and physiological, biomechanical, and perceptual parameters. The starting speed of the treadmill for the 3-km TT was set to 90% of the recent 5-km time and an incline of 1%. All runners were instructed to run the 3-km TT as fast as possible. Before the start of the 3-km TT, all runners warmed-up for 10 min at 10 km/h and additional 60 s corresponding to the starting speed of the 3-km TT. After the warm-up all rested for 3 min. Treadmill speed changes requested by each runner during the 3-km TT were indicated by hand signal (thumb up = 0.5 km/h speed increase; thumb down = 0.5 km/h speed decrease) and adjusted manually by an investigator. The investigator verbally communicated the covered distance to the runners in 500 m segments up to 2,500 m and in 100 m segments during the last 500 m. No verbal encouragement was provided. The time to complete the 3-km TT was measured with a stopwatch (PC-90, schütt-sport©, Marburg, Germany). During the 3-km TT, HR (Polar Vantage M2, Polar Electro© Oy, Kempele, Finland) was continuously measured and averaged over the entire 3-km TT. Before the start, at completion, and 1 and 3 min after the 3-km TT capillary blood samples were taken from the right earlobe and immediately analyzed (Lactate Pro 2, Arkray KDK, Japan) to measure blood lactate concentration. After the 3-km TT all runners rated their perceived exertion (RPE) with the 6–20 Borg scale (21). During the 3-km TT, stride frequency and stride length were continuously analyzed with the Polar Stride Sensor (Polar Stride, Polar Electro© Oy, Kempele, Finland) attached to the left running shoe. Additionally, all runners rated their perceived shoe comfort and shoe performance directly after the 3-km TT (8).

Previous research demonstrates a high to very high reliability of 1,500-m TT, 3.2-km TT (25), and 5-km TT (26, 27, 22) with well-trained runners on the treadmill.

### 2.6 Statistical analysis

Descriptive statistics of the experimental data are reported as mean ± SD. All data were confirmed to be normally distributed by the Shapiro-Wilk test, and the Levene test confirmed the homogeneity of variance, so no further transformation was required.

Differences between the data of trials with carbon fiber and control insoles were assessed by applying the student's *t*-test for paired samples and effect size Cohen's d (*d*) (difference between the means/pooled standard deviation) (28). The small, moderate, and large effect size thresholds were 0.20, 0.50, and 0.80, respectively (28). The level of significance was set *a priori* to *p* ≤ 0.05. All analyses were performed with SPSS 27.0 software (SPSS Inc., Chicago, IL, USA).

## 3 Results

### 3.1 Incremental treadmill step test

**Figure 3:**
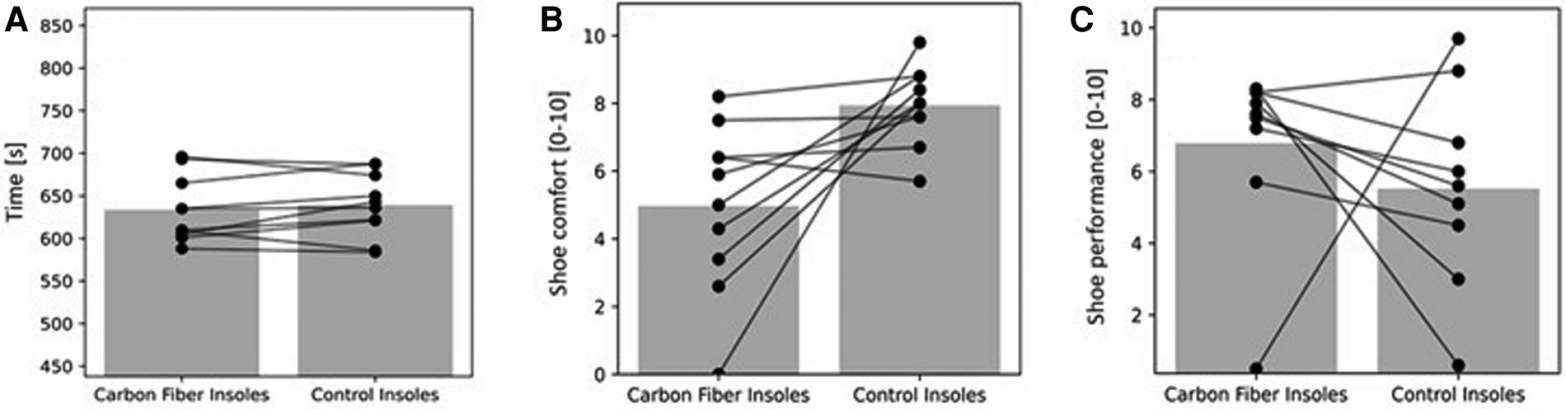
(**A**) time to exhaustion; (**B**) shoe comfort; (**C**) shoe performance of the 10 runners during incremental treadmill testing. The bar represents the mean values of 10 runners. Circles joined by lines represent the individual data of each runner for carbon fiber and control insoles.

Table 4 summarizes the results as mean values from all participants for the submaximal running velocities (i.e., 10, 12, 14, and 16 km/h). The data for 18 km/h are not included since three out of ten runners did not complete the 18 km/h increment. Figure 3 shows individual data of the ten runners for the parameters time to exhaustion, shoe comfort and shoe performance during incremental treadmill testing in both conditions. Time to exhaustion, oxygen consumption, respiratory exchange ratio, blood lactate concentration, and heart rate during the incremental treadmill test showed no significant differences between the two conditions (Table 4 and Figure 3). Also, the ratings of perceived exertion, stride frequency, and stride length demonstrated no significant differences between the two conditions (Table 4). Although, perceived shoe performance during running was not significantly different between the two conditions, the perceived shoe comfort during running was rated not as high with the carbon fiber insoles compared to control insoles (Table 4 and Figure 3).

**Table 4:** All variables (mean ± standard deviation) during incremental treadmill test of the 10 runners running with carbon fiber insoles and the control insoles.

Variable	Running speed	Carbon fiber insole	Control insole	%*Δ*	*p*	*d*
Oxygen consumption [ml/kg/min]	10 km/h	35.2 ± 2.1	33.3 ± 6.2	5.7	.504	.220
	12 km/h	41.9 ± 2.5	40.1 ± 4.3	4.5	.282	.385
	14 km/h	48.0 ± 2.8	46.4 ± 3.4	3.4	.273	.392
	16 km/h	54.0 ± 3.5	52.5 ± 3.5	2.9	.418	.284
Respiratory exchange ratio [a.u.]	10 km/h	0.81 ± 0.03	0.80 ± 0.03	1.3	.656	.146
	12 km/h	0.86 ± 0.03	0.86 ± 0.02	0.0	.810	−.083
	14 km/h	0.89 ± 0.03	0.89 ± 0.03	0.0	.751	−.109
	16 km/h	0.92 ± 0.03	0.92 ± 0.03	0.0	.325	.374
Blood lactate concentration [mmol/L]	10 km/h	2.4 ± 0.5	2.4 ± 0.6	0.0	.754	−.102
	12 km/h	2.3 ± 0.9	2.6 ± 0.8	−115	.480	−.247
	14 km/h	3.0 ± 0.7	3.4 ± 0.7	−11.8	.213	−.451
	16 km/h	5.9 ± 1.7	6.7 ± 1.9	−11.9	.188	−.479
Heart rate [bpm]	10 km/h	132 ± 7	130 ± 6	1.5	.353	.310
	12 km/h	148 ± 5	146 ± 6	1.4	.343	.316
	14 km/h	162 ± 5	161 ± 5	0.6	.507	.219
	16 km/h	174 ± 4	173 ± 6	0.6	.672	.138
RPE [6–20]	10 km/h	7.9 ± 1.2	8.3 ± 1.2	−4.8	.373	−.296
	12 km/h	11.4 ± 1.2	11.2 ± 1.3	1.8	.443	.254
	14 km/h	14.2 ± 1.0	14.3 ± 0.9	−0.7	.758	−.101
	16 km/h	17.0 ± 0.7	17.1 ± 0.7	−0.6	.758	−.101
Stride frequency [1/min]	10 km/h	80.4 ± 6.4	80.3 ± 6.4	0.1	.898	.042
	12 km/h	82.3 ± 5.6	82.1 ± 5.2	0.2	.669	.140
	14 km/h	84.6 ± 4.9	84.3 ± 4.2	0.4	.521	.211
	16 km/h	79.8 ± 22.9	79.4 ± 23.2	0.5	.387	.288
Stride length [cm]	10 km/h	194.4 ± 37.8	201.6 ± 31.0	−3.6	.693	−.136
	12 km/h	229.4 ± 35.8	230.4 ± 30.0	−0.4	.641	.161
	14 km/h	257.2 ± 32.8	255.6 ± 28.6	0.6	.309	.362
	16 km/h	266.2 ± 43.0	267.6 ± 43.6	−0.5	.913	−.037
Time to exhaustion [min:s]	–	25:13 ± 2:31	25:15 ± 2:12	−0.1	.948	−.021
Shoe performance [0–10]	–	6.4 ± 1.8	5.7 ± 2.2	12.3	.531	.206
Shoe comfort [0–10]	–	3.3 ± 2.1	7.9 ± 1.2	−58.2	.001*	−1.646

%*Δ* = percentual difference between the mean values of carbon fiber insoles vs. control insoles. RPE, rates of perceived exertion; bpm, beats per minute.

* Significant difference between running with carbon fiber insoles and control insoles (*p* ≤ 0.05).

### 3.2 3-km time-trial on the treadmill

**Figure 4:**
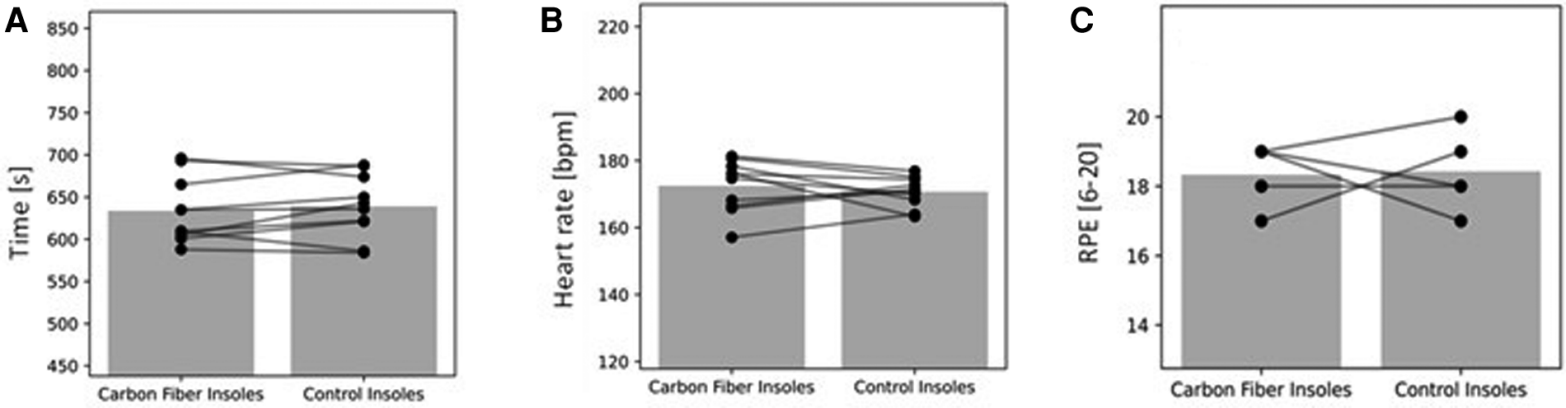
(**A**) in the 3-km TT; (**B**) mean heart rate during the 3-km TT; (**C**) rating of perceived exertion during the 3-km TT of the ten runners. The bars represent mean values of the 10 runners. The circles joined by lines represent individual data of each runner.

**Figure 5:**
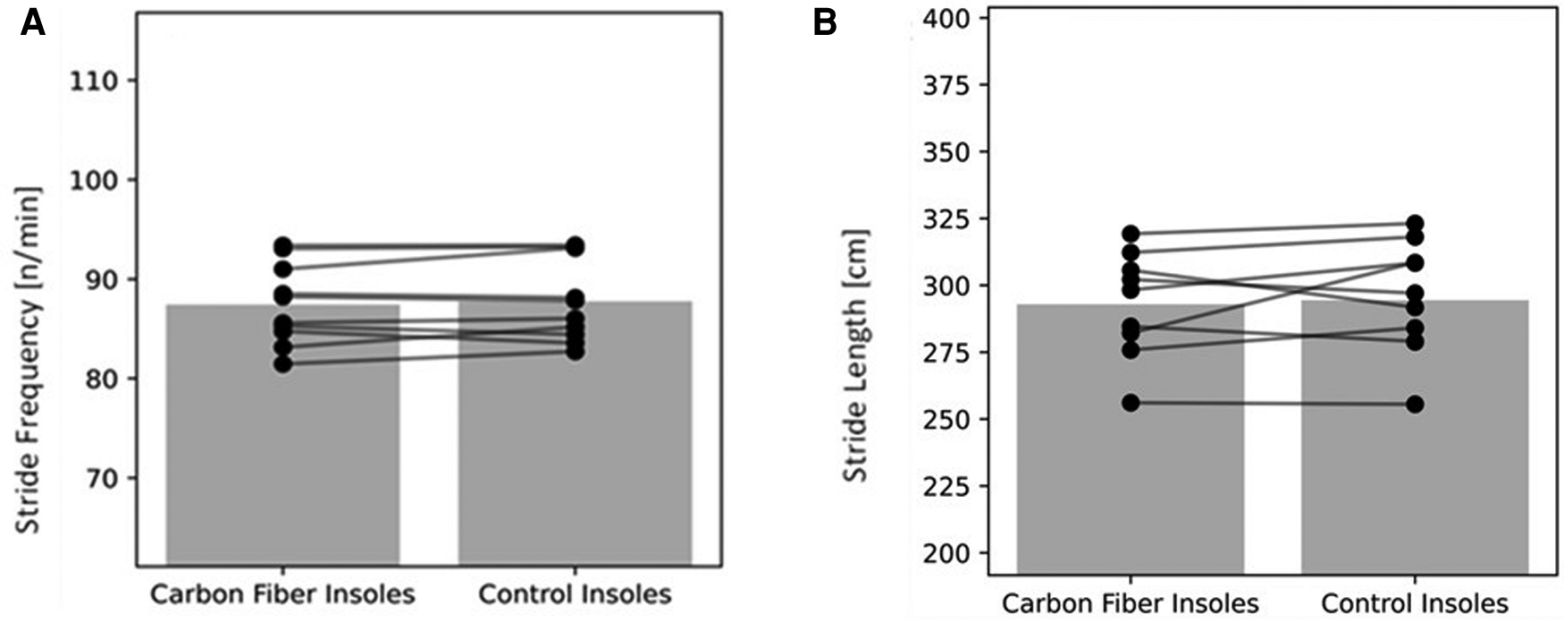
(**A**) stride frequency during 3-km TT; (**B**) stride length during 3-km TT in the ten runners. The bars represent mean values of the ten runners. Circles joined by lines represent individual data of each runner.

**Figure 6:**
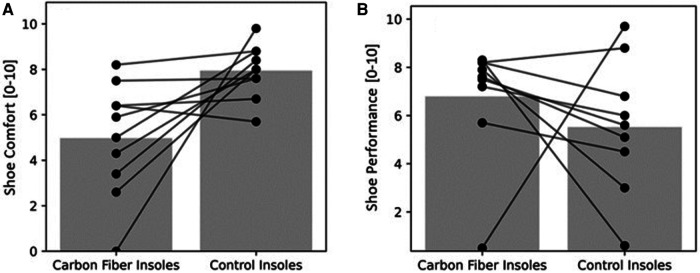
(**A**) shoe comfort; and (**B**) shoe performance during 3-km TT in the ten runners. The bars represent mean values of 10 runners. Circles joined by lines represent individual data of each runner.

Results for the 3-km time trial during the treadmill are presented in Table 5 and Figures 4–6.

**Table 5:** All variables (mean ± standard deviation) obtained during and after the 3-km TT test of the ten runners running with carbon fiber insoles and control insoles.

Variable	Carbon fiber insoles	Control insoles	%*Δ*	*p*	*d*
3-km time [min:s]	10:34 ± 0:37	10:39 ± 0:35	−0.8	.410	−.273
Heart rate [bpm]	172.5 ± 7.4	170.7 ± 4.3	1.1	.441	.255
Blood lactate concentration [mmol/L]	9.5 ± 3.7	11.0 ± 4.2	−13.6	.200	−.501
RPE [6–20]	18.3 ± 0.7	18.4 ± 0.9	−0.5	1.000	.000
Stride frequency [1/min]	87.4 ± 3.9	87.8 ± 3.9	−0.5	.389	−.286
Stride length [cm]	293.2 ± 17.8	294.4 ± 19.8	−0.4	.764	−.098
Shoe performance [0–10]	6.8 ± 2.3	5.5 ± 2.5	23.6	.416	.269
Shoe comfort [0–10]	5.0 ± 2.3	7.9 ± 1.1	−36.7	.017	−.919

%*Δ* = percentual difference between the mean values of carbon fiber insoles vs. control insoles. RPE, rates of perceived exertion; bpm, beats per minute.

The 3-km time and all parameters measured during the 3-km time trial did not differ significantly between the two conditions, except for lower shoe comfort while running with the carbon fiber compared to the control insoles (Table 5 and Figures 4–6).

## 4 Discussion

The present study aimed to compare running economy and maximal running performance as well as physiological, biomechanical, and perceptual parameters at submaximal and maximal running speeds during an incremental treadmill test and during a 3-km time-trial in male trained runners wearing either a pair of downwards curved carbon fiber insoles or a pair of control insoles in road racing shoes.

The main results comparing downwards curved carbon fiber insoles with control insoles are as follows: (i) running economy did not differ significantly; (ii) maximal running performance in the incremental treadmill test and the 3-km TT showed no significant differences; (iii) all physiological, biomechanical and most of perceptual parameters measured during and following the incremental treadmill test and in the 3-km TT did not differ significantly; (iv) shoe comfort during the incremental treadmill test and the 3-km TT was higher while running in control insoles compared to the downwards curved carbon fiber insoles.

Recent research demonstrated that shoes with novel stiffening technology, e.g., integrated upward curved carbon fiber plates and novel lightweight and resilient foam materials like Pebax (polyether block amide) in the midsole, could improve laboratory-based running economy (8, 6, 5, 7). In some cases, the improved running economy translated to enhanced running performance on the treadmill (8). Meta-analytical data confirmed improvements in running economy when running with shoes with embedded carbon fiber plates and novel Pebax foam in the midsole (14).

Contrasting previous studies (8, 6, 5), our analysis found no significant physiological or biomechanical differences between the two conditions. While previous research (8, 5–7) reported improved running economy with advanced shoe technology, our participants showed a non-significant increase in oxygen consumption (2.9%–5.7%) with downward curved carbon fiber insoles. This observation, despite its statistical insignificance, might be relevant in elite sports, where marginal differences can be crucial. The slight increase in submaximal oxygen consumption did not negatively impact performance in the 3-km TT and incremental treadmill tests, suggesting that the practical impact of shoe technology might be nuanced and warrants further investigation.

Several reason could account for the similar properties between the downwards curved carbon fiber insoles compared with control insoles:

(i) Since the carbon fiber insoles and the control insoles were matched by mass in the present study, a difference in shoe mass is not a valid explanation of the absent improvements in running economy. (ii) majority of studies demonstrating benefits of carbon fiber plates examined shoes with permanently integrated carbon fiber plates. Therefore, a potential “integration effect” or interaction between carbon fiber plate and midsole of the running shoe should be considered. A recent study demonstrated that the observed benefits of a novel running shoe technology are most likely not caused by the carbon fiber plate alone. Instead, improved running economy might stem from a combination and interaction of the novel midsole Pebax foam, the geometry of the running shoe, and the embedded carbon fiber plate (29). To test this assumption, Healey and Hoogkamer (29) applied 6 medio-lateral cuts through the carbon fiber plate in the forefoot region of the Nike Vaporfly 4% shoe (Nike Inc., Beaverton, OR, USA) to reduce the longitudinal bending stiffness of the carbon fiber plate. The 6-time cut carbon fiber plate in the shoe caused no significant impairment (mean ± SD 0.55% ± 1.77%) of running economy compared to the shoe with the intact carbon fiber plate. Consequently, the authors concluded that curved carbon fiber plates are not solely accountable for improvements in running economy but rather the resilient Pebax midsole foam, shoe geometry, and other effects of the curved carbon fiber plate, which are unrelated to the longitudinal bending stiffness. This hypothesis is supported by an earlier study showing a 87% superior energy return of a midsole made of Pebax foam in comparison to midsoles made of lightweight ethylene-vinyl acetate foam and thermoplastic polyurethane foam (5). The suggestion that carbon fiber plates in the shoe are not solely responsible for improvements in running economy is supported by Beck et al. They demonstrated that improving the longitudinal bending stiffness of a running shoe with the assistance of carbon fiber plates does not improve running economy (30). And even earlier studies showed that increasing the longitudinal stiffness of the running shoe with a carbon fiber plate alone has a limited impact on the running economy of ∼1% (19). However, the present study was the first to analyze the impact of an insertable downwards curved carbon fiber insole on running economy and maximal running performance during treadmill running. The present study demonstrated that neither running economy nor maximal running performance was improved with downwards curved carbon fiber insoles in a conventional road running shoe without Pebax foam in the midsole. This leads to the conclusion that a carbon fiber plate with a downward curvature inserted in a shoe without PEBAX material in the midsole has no beneficial effects on running performance or physiological parameters. It is possible that the advantageous energy-returning properties of the midsole material, as well as the quantity, a height of up to 4 cm, in novel running shoes represents an important factor for improvements in running economy (5, 29). The function of the carbon fiber plates in novel running shoes could be to spread the force distribution more equally during the impact to load the midsole foam better with energy. Another possible explanation of beneficial effects of embedded carbon fiber plates is the teeter-totter-effect resulting from the stiffness and the curvature of the carbon fiber plate (31, 32). However, up to date no experimental data exists to directly support the teeter-totter effect and the supposedly optimized force distribution to the midsole foam with the help of a carbon fiber plate.

A systematic review with meta-analysis (14) emphasized a positive correlation between the degree of longitudinal bending stiffness and improvements in running economy. It seems likely that the carbon fiber insoles employed in the present study did not possess enough longitudinal bending stiffness to have a robust impact on the running economy. Other studies suggest that optimal bending stiffness levels are individual (33, 19) and speed-dependent (34, 35). Therefore, a carbon insole with a single absolute bending behavior might not be suitable to improve running economy and performance in a group of runners with different strike patterns and body mass. Additionally, the hypothesis was that a downward curved carbon fiber insole might induce an earlier bending of the carbon element and thus induce a beneficial change of the amount and timing of energy storage and return at the metatarsophalangeal joints. It is likely that the carbon fiber insoles employed in the present study did not demonstrate any positive effects due to their insufficient longitudinal bending stiffness, despite the potential positive downward curvature.

In the present study, we employed insertable, in contrast to midsole-embedded, carbon fiber plates as an insole in a racing shoe with a stack height of only 18.0 mm (racing flats) without any novel and highly resilient foam materials, like Pebax. The Saucony fastwitch 9 running shoe's midsole consists of an EVA foam exhibiting a distinct inferior energy return in comparison to Pebax. Since we could not detect significant physiological and biomechanical differences between the downwards curved carbon fiber compared to control insoles [which is in contrast to previous findings (5–8)] we assume, also in light of Healey and Hoogkamer (29) suggestion, that the lack in difference in running economy may have resulted from the absent combination of novel and resilient midsole foam materials, shoe geometry, and effects of the downwards curved carbon fiber plate.

Interestingly, in the present investigation shoe comfort was impaired when running with the downwards curved carbon fiber compared to control insoles during the incremental treadmill testing as well as during the 3-km time trial. The reasons for the impaired shoe comfort could be attributed to the fact that the participants ran with direct contact to the carbon fiber insole when inserted into the shoe. Hence, runners had direct foot contact with the carbon fiber insole during each stance phase without any further cushioning.

### 4.1 Limitations

The present results may be different when the carbon fiber insoles are worn in different running shoes, especially when the carbon fiber insoles are combined with a midsole made of more resilient foam.

The primary objective of this study was to assess the impact of insertable carbon fiber insoles on running economy, running performance, and various physiological, biomechanical, and perceptual variables during treadmill running in a controlled laboratory setting. Although the findings revealed no significant effects on most of the measured parameters, it is important to underscore the limited ecological validity of the present study. In the context of real-life long-distance running, factors such as intense central and/or peripheral fatigue may activate mechanisms that could potentially impact the outcomes.

## 5 Conclusion

The present analysis did not reveal any significant or practically relevant positive impact on running economy, running performance, and selected physiological, biomechanical, and perceptual parameters when running with downwards curved carbon fiber insoles compared to control insoles. From a practical perspective, the results suggest that this downwards curved carbon fiber insoles in a conventional road running shoe do not improve performance.

## Data Availability

The original contributions presented in the study are included in the article/Supplementary Material, further inquiries can be directed to the corresponding author.
