# Supplementary material for: Acute physiological, biomechanical, and perceptual responses of runners wearing downward-curved carbon fiber insoles
Source: Front Sports Act Living. 2024 Apr 5;6:1340154. doi: 10.3389/fspor.2024.1340154 (PMC11026664; doi:10.3389/fspor.2024.1340154)
Supplement: Supplementary file 1 [file Datasheet1.pdf]

Julius-Maximilians-Universität Würzburg
Fakultät für Humanwissenschaften
Ethikkommission für den Master of Science Exercise Science & Training

Julius-Maximilians-Universität Würzburg
Faculty of Human Sciences
Ethical Review Board of the Master Program Exercise Science and Training

To whom it may concern

Reference: Ethical Approval Statement of the Ethical Review Board of the Master Program Exercise Science and Training

The ethical review board herewith confirms the ethical approval to the application of PD Dr. Florian Engel and allocates the following number to the ethical approval:

EV2023/1-13.06.2023

**EV**	**2023**	**1**	**13.06.2023**
**Ethical vote**	**Year**	**Running number of applications**	**Date of the ethical vote**

Betty Spalich

Institut für Sportwissenschaft
der Universität Würzburg
Judenbühlweg 11
97082 Würzburg

03.07.2023
